# SPHARA - A Generalized Spatial Fourier Analysis for Multi-Sensor Systems with Non-Uniformly Arranged Sensors: Application to EEG

**DOI:** 10.1371/journal.pone.0121741

**Published:** 2015-04-17

**Authors:** Uwe Graichen, Roland Eichardt, Patrique Fiedler, Daniel Strohmeier, Frank Zanow, Jens Haueisen

**Affiliations:** 1 Institute of Biomedical Engineering and Informatics, Faculty of Computer Science and Automation, Technische Universität Ilmenau, Ilmenau, Germany; 2 eemagine GmbH, Berlin, Germany; 3 Biomagnetic Center, Department of Neurology, University Clinic Jena, Jena, Germany; University of Amsterdam, NETHERLANDS

## Abstract

Important requirements for the analysis of multichannel EEG data are efficient techniques for signal enhancement, signal decomposition, feature extraction, and dimensionality reduction. We propose a new approach for spatial harmonic analysis (SPHARA) that extends the classical spatial Fourier analysis to EEG sensors positioned non-uniformly on the surface of the head. The proposed method is based on the eigenanalysis of the discrete Laplace-Beltrami operator defined on a triangular mesh. We present several ways to discretize the continuous Laplace-Beltrami operator and compare the properties of the resulting basis functions computed using these discretization methods. We apply SPHARA to somatosensory evoked potential data from eleven volunteers and demonstrate the ability of the method for spatial data decomposition, dimensionality reduction and noise suppression. When employing SPHARA for dimensionality reduction, a significantly more compact representation can be achieved using the FEM approach, compared to the other discretization methods. Using FEM, to recover 95% and 99% of the total energy of the EEG data, on average only 35% and 58% of the coefficients are necessary. The capability of SPHARA for noise suppression is shown using artificial data. We conclude that SPHARA can be used for spatial harmonic analysis of multi-sensor data at arbitrary positions and can be utilized in a variety of other applications.

## Introduction

The discrete Fourier analysis of 2D data defined on a flat surface and represented by a Cartesian or a regular grid is very common in digital image processing and a fundamental tool in many applications. For such data, the basis functions (BF) for the Fourier transformation are usually implicitly specified in the transformation rule, compare [1].

In many applications, however, the sensors for data acquisition are not located on a flat surface and can not be represented by Cartesian or regular grids. One important example for non-regular sensor positions is the application of electroencephalography (EEG). In EEG, the sensors are placed at predetermined positions on a surface in space ℝ^3^, the head shape. The positions of the sensors in these systems can be described by means of triangular meshes. Because of the particular sensor arrangement, the spatial analysis of multi-sensor data can not be performed using a standard 2D Fourier analysis.

Several methodologies have been proposed previously that are applicable for signal enhancement, feature selection and dimensionality reduction of multichannel EEG data. Examples are the principal component analysis (PCA), the independent component analysis (ICA), the parallel factor analysis (PARAFAC) and matching pursuit (MP) using Bessel or multichannel Gabor atoms.

PCA, also known as Karhunen-Loève transform, is a statistical procedure commonly used to reduce a large number of variables to a smaller number of principal components (PC). These components are pairwise orthogonal and describe the maximum amount of variance in the original multivariate data. PC are determined by computing the eigensystem of the covariance matrix of the measured data. PCA is used to decompose multichannel EEG data into orthogonal components with respect to their spatial distribution. PCA is applied for signal enhancement, feature selection and dimensionality reduction in BCI [2–4], for source localization [5, 6] and for artifact recognition and removal [7]. However, the basis functions of the PCA are determined by using the measured EEG data. Thus, PCA can be used only after a certain number of time samples are recorded.

Another linear decomposition technique is ICA. In contrast to PCA, the ICA approach maximizes the statistical independence between the signal components (independent components). Furthermore, basis vectors of the ICA are in general not orthogonal and there is no closed form expression to calculate the components. Therefore, iterative approaches are used to determine the individual components, which results in higher computational costs. One application of ICA is the separation of brain activity from artifacts. It is assumed that brain activity and artifacts are statistically independent processes, which result in statistically independent components of the measured signal [8–11]. Another application of ICA is the decomposition of evoked fields or potentials [12, 13]. Furthermore, ICA is employed to separate spatially overlapping EEG activities [10]. In BCI applications, ICA is used for signal enhancement and feature selection [3, 14, 15]. Like PCA, ICA can be used only after a certain number of time samples is recorded.

Both PCA and ICA are used to decompose two-way data. Usually they are employed to perform a space-time decomposition of multichannel EEG signals. Using parallel factor analysis (PARAFAC), also known as canonical (polyadic) decomposition, a multi-way decomposition of multichannel EEG data is possible. By employing PARAFAC, wavelet transformed multichannel EEG signals can be decomposed into channel, frequency and time components [16]. In another application of PARAFAC, a five-way analysis of EEG data is performed and the recorded data are decomposed into channel, frequency, time, subject and condition components [17]. PARAFAC requires a high computational effort. Further, preprocessing steps, such as a time-frequency decomposition, are often necessary. In BCI, PARAFAC is used for feature selection [18].

The Matching Pursuit method is used to decompose single channel EEG signals into time-frequency components using an over-complete dictionary [19]. There also exists an extended version of the matching pursuit method for the application to multichannel EEG data using multichannel Gabor atoms [20, 21]. In addition, Bessel atoms are used for the spatial decomposition of MEG data [22]. When using Bessel atoms, the basis functions are determined prior to data recording. However, discretized Bessel functions are only applicable for the spatial decomposition of the data of certain sensor configurations; otherwise, the required orthogonality of the Bessel functions is lost. As part of the data decomposition, a very large overdetermined dictionary with basis functions has to be searched repeatedly.

Another approach for spatial analysis of EEG data is the use of spherical harmonics, where the head shape is approximated by a spherical surface [23]. The effect of inevitable mapping errors that arise in this approach on the spatial spectrum is difficult to estimate. Moreover, due to the anatomy of the head, only parts of the spherical surface can be sampled in EEG, therefore, they must be combined with window functions.

Laplace spectra, which we use for the spatial decomposition, are widely applied in the field of graph theory [24] and computer graphics [25–27]. A similar approach, called Spherical Cap Harmonic Analysis is used in geosciences to analyze the earth magnetic field [28, 29].

Surface Laplacian approaches are applied in EEG to improve the spatial resolution [30–32]. Laplacian eigenmaps are also deployed for EEG studies [33, 34] to investigate EEG time series and topographies. However, the Laplacian eigenbasis of these two approaches is not taking into account the spatial arrangement of the EEG electrodes in ℝ^3^.

In this publication we present a new method for spatial harmonic analysis (Sphara) of EEG data using the eigenbasis of the Laplace-Beltrami operator of the meshed surface of electrode positions. Using this approach, BF of spatial harmonics for arbitrary arrangements of EEG electrodes can be generated. The recorded EEG data are decomposed by projection into the space of the BF. In addition, a number of possible applications are presented, such as analysis of the spatial properties of SEP data, dimensionality reduction and noise reduction.

We presented preliminary research results in the field of spatial harmonic analysis of multi-sensor in a talk at the 8th International Symposium on Noninvasive Functional Source Imaging of the Brain and Heart & International Conference on Bioelectromagnetism – NFSI & ICBEM, 2011, Banff [35].

## Materials and Methods

### Overview

In this section, the new method for the spatial harmonic analysis of EEG data is derived. Starting from the well-investigated continuous case, we introduce several discretization schemes, which can be applied to EEG sensor arrangements. The BF, which are used for the spatial signal decomposition, are determined by solving the eigenvalue problem for the Laplace-Beltrami operator, which is also referred to as the Helmholtz equation. We show relevant properties of the BF that are necessary for the application in a signal processing frameworks. Finally, computational complexity and algorithmic aspects are considered.

### Continuous case

#### Continuous Laplace-Beltrami Operator

We assume a compact Riemannian manifold (𝓜, *g*) of dimension *m*, where 𝓜 is a connected manifold and real-differentiable of class *C* ^∞^. The function *g* defines for each point *p* ∈ 𝓜 the inner product of the tangent space *T*_*p*_𝓜. The union of all tangent spaces of 𝓜 is *T*𝓜. An *m*-dimensional manifold is a topological space, that locally resembles the Euclidean space ℝ^*m*^. Within this paper, we focus on two-dimensional manifolds representing surfaces in ℝ^3^.

If the manifold 𝓜 has a boundary *B* = ∂𝓜, it is assumed that 𝓜 is oriented and that *C* ^∞^ also applies to *B*. The outward unit normal vector field on *B* is denoted by *n*.

We consider a real-valued function *f* with *f* ∈ *L* ^2^(𝓜, *g*) and *f* is *C* ^*k*^ with *k* ≥ 2. The directional derivative of *f* at *p* ∈ 𝓜 for each *ξ* ∈ *T*_*p*_𝓜 is denoted by *ξf*. The gradient of *f* is the vector field on 𝓜 with

$$\begin{array}{c} \mathrm{grad}f=\xi f\;\text{for}\;\text{all}\;\xi \in Tℳ\; \end{array}.$$ (1)

Let *X*, *Y* be vector fields, which are *C* ^*k*^ with *k* ≥ 1, and let ∇_*ξ*_ *X* be the covariant derivative of *X* with respect to *ξ* for all *p* ∈ 𝓜 and *ξ* ∈ *T*_*p*_𝓜. And ∇_*ξ*_ additionally fulfills ∇_*ξ*_(*X* + *Y*) = ∇_*ξ*_ *X* + ∇_*ξ*_ *Y* and ∇_*ξ*_(*fX*) = (*ξf*)*X*(*p*) + *f*(*p*)∇_*ξ*_ *X*. The divergence of *X* is defined by

$$\begin{array}{c} \mathrm{div}X=\mathrm{trace}(\nabla_{\xi }X)\;, \end{array}$$ (2)

where *ξ* ranges over *T*_*p*_𝓜. The continuous Laplace-Beltrami operator Δ is defined by

$$\begin{array}{c} \Delta f=\mathrm{div}(\mathrm{grad}f)\; \end{array}.$$ (3)

For a more extensive derivation of the continuous Laplace-Beltrami operator see for example [36, 37].

If the manifold 𝓜 possesses a boundary *B*, several boundary conditions (BC) can be applied

$$\begin{array}{ccc} f=0, & \;\text{on}\;\partial ℳ & \text{Dirichlet}\;\text{BC} \end{array}$$ (4)

$$\begin{array}{ccc} \frac{\partial f}{\partial n}=n\cdot \nabla f=0, & \;\text{on}\;\partial ℳ & \text{Neumann}\;\text{BC} \end{array}$$ (5)

$$\begin{array}{ccc} \eta \;\frac{\partial f}{\partial n}+\zeta \;f=0, & \;\text{on}\;\partial ℳ & \text{Robin}\;\text{(mixed)}\;\text{BC} \end{array}$$ (6)

with the nabla operator ∇ and *η* ≠ 0, *ζ* ≠ 0. The utilized boundary condition affects the solution of Eq (7) and thus the properties of the determined basic functions. When using Dirichlet BC, the values of functions *f* on the boundary *B* of the manifold 𝓜 are set to 0. For Neumann BC, the normal derivative of the function *f* on the boundary *B* of the manifold 𝓜 is fixed to 0. The Robin BC is a linear combination of Dirichlet and Neumann BC using the coefficients *η* and *ζ*. In our application, the manifold 𝓜 describes the area of the head, which is covered by the EEG sensor system. The potential field of the head surface extends beyond the range that is covered by 𝓜. The potentials on the boundary *B* of the manifold is generally not 0 or constant. Therefore, the Neumann BC is more appropriate to compute the basis functions using the presented approach.

#### Properties of Continuous Laplace-Beltrami Operators and their Eigensystems

The operator Δ possesses a number of properties that enable the computation of harmonic BF by solving the Laplacian eigenvalue problem

$$\begin{array}{c} \Delta \Phi_{i}=\lambda_{i}\Phi_{i} \end{array}.$$ (7)

These properties are: Δ*f* = 0 for constant *f*, symmetry, local support, linear precision, maximum principle, and positive semi-definiteness [38]. A basis for a harmonic analysis of functions, which are defined on the Riemannian manifold, can be determined by finding all eigenvalues *λ*_*i*_ ∈ ℝ and the associated non-trivial eigenfunctions *ϕ*_*i*_, with *ϕ*_*i*_ ∈ *C* ^2^(𝓜). The set of all eigenvalues ${\left\{\lambda_{i}\right\}}_{i=1}^{\infty }$ defines the spectrum of 𝓜

$$\begin{array}{c} spec\left(ℳ\right)={\left\{\lambda_{i}\right\}}_{i=1}^{\infty }=\left\{0\leq \lambda_{1}<\lambda_{2}<\ldots <\lambda_{\infty }\right\}, \end{array}$$ (8)

with lim_*i* → ∞_ *λ*_*i*_ → ∞. The set of the eigenfunctions ${\left\{\phi_{i}\right\}}_{i=1}^{\infty }$ forms an orthonormal basis and can be used for the spectral analysis of functions defined on 𝓜. The eigensystem of the Laplace-Beltrami operator can be considered as basis of a generalized Fourier analysis on 𝓜, see also [36, 37, 39]. The continuous approach to determine the BF for 𝓜 will be referred to as CONT later in the text.

**Fig 1:**
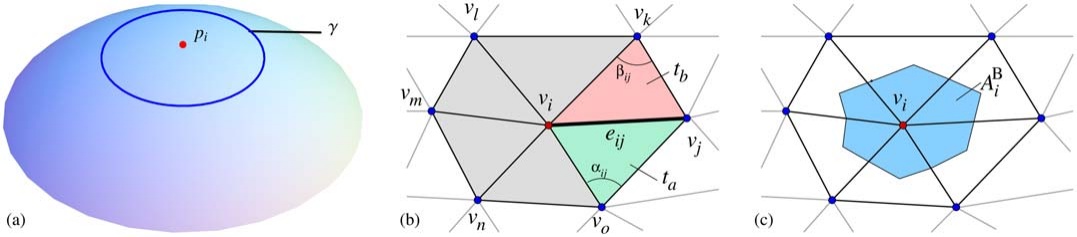
The approximation of the Laplace-Beltrami operator using Eq (9). (a) continuous representation; (b) discrete representation. The neighborhood *i* ^⋆^ of vertex *v*_*i*_ consists of the vertices {*v*_*x*_ ∈ *V* : *e*_*ix*_ ∈ *E*}. Either the length of *e*_*ij*_ or the size of the two angles *α*_*ij*_ and *β*_*ij*_ opposed to the edge *e*_*ij*_ are used to estimate the weight *w*(*i*, *j*) for *e*_*ij*_. The two triangles *t*_*a*_ and *t*_*b*_ both share the edge *e*_*ij*_; (c) the area of the barycell $A_{i}^{\text{B}}$ for the vertex *v*_*i*_.

For any point *p*_*i*_ on 𝓜, an approximation Δ_*A*_ for Δ is given by the curvilinear integral

$$\begin{array}{c} \Delta_{A}f\left(p_{i}\right)=\frac{1}{\left|\gamma \right|}\int_{p\in \gamma }(f\left(p_{i}\right)-f\left(p\right))\text{d}p\;, \end{array}$$ (9)

where *γ* is a closed simple curve on 𝓜 surrounding the point *p*_*i*_, and ∣*γ*∣ is the length of *γ* [40, 41], see Fig 1(a). In practical applications, only a few discrete points of *γ* are usually known. In the recording of EEG data, the continuous function *f* describing the potential distribution on the surface of the head is sampled only at the electrode positions. For this reason, discretization approaches for Δ are necessary. If a geometric discretization approach is used, the discrete Laplace-Beltrami operator converges to the continuous form Δ_*D*_ → Δ when the grid is refined [38].

### Discrete case

#### Discrete representation of surfaces

For the discrete case we assume that the sensors are located on an arbitrary surface, which is represented by a triangular mesh in ℝ^3^. The mesh *M* = {*V*, *E*, *T*} consists of vertices *v* ∈ *V*, edges *e* ∈ *E* and triangles *t* ∈ *T*. Each vertex *v*_*i*_ ∈ ℝ^3^ represents a sensor position. The number of vertices, edges and triangles of *M* are defined by ∣*V*∣, ∣*E*∣ and ∣*T*∣, respectively. The neighborhood *i* ^⋆^ for a vertex *v*_*i*_ ∈ *V* is defined by *i* ^⋆^ = {*v*_*x*_ ∈ *V* : *e*_*ix*_ ∈ *E*}, see Fig 1(b). The number of neighbors of *v*_*i*_ is *n*_*i*_ = ∣*i* ^⋆^∣. The angles *α*_*ij*_ and *β*_*ij*_ are located opposed to the edge *e*_*ij*_. The triangles *t*_*a*_ and *t*_*b*_, defined by the vertices (*v*_*i*_, *v*_*j*_, *v*_*o*_) and (*v*_*i*_, *v*_*k*_, *v*_*j*_), share the edge *e*_*ij*_. The set of triangles sharing the vertex *v*_*i*_ is given by *i* ^▿^ = {*t*_*x*_ ∈ *T* : *v*_*i*_ ∈ *t*_*x*_}. The area of a triangle *t* is given by ∣*t*∣. An example for these mesh components is illustrated in Fig 1(b).

#### Discrete Laplace-Beltrami Operators

A function $\vec{f}$ is defined for all vertices *v*_*i*_ ∈ *V*, it applies $\vec{f}:v_{i}\to R$ with *i* = 1, …, ∣*V*∣. A discretization Δ_*D*_ of the continuous Laplace-Beltrami operator in Eq (9) for $\vec{f}$ is given by

$$\begin{array}{c} \Delta_{D}{\vec{f}}_{i}=b_{i}^{-1}\sum_{x\in i^{⋆}}w\left(i,x\right)({\vec{f}}_{i}-{\vec{f}}_{x})\;, \end{array}$$ (10)

with the weighting function *w*(*i*, *x*) for edges *e*_*ix*_ ∈ *E* and the normalization coefficient *b*_*i*_ for the vertex *v*_*i*_. For practical applications it is convenient to transform Eq (10) into matrix notation. The elements of the matrices *B* ^−1^ and *S* are determined using the coefficients *b*_*i*_ and *w*(*i*, *x*) of Eq (10). *B* ^−1^ is a diagonal matrix, the elements are

$$\begin{array}{c} B_{ij}^{-1}=\{\begin{array}{cc} b_{i}^{-1} & \text{if}\;i=j\\ 0 & \text{otherwise}\;, \end{array} \end{array}$$ (11)

and the entries of *S* are

$$\begin{array}{c} S_{ij}=\{\begin{array}{cc} \sum_{x\in i^{⋆}}w\left(i,x\right) & \text{if}\;i=j\\ -w(i,x) & \text{if}\;e_{ix}\in E\\ 0 & \text{otherwise}\;. \end{array} \end{array}$$ (12)

A Laplacian matrix *L* can be expressed as product of a diagonal matrix *B* ^−1^ and a matrix *S*

$$\begin{array}{c} L=B^{-1}\;S\;, \end{array}$$ (13)

compare also [42]. The size of the Laplacian matrix *L* for a mesh *M* is *n* × *n*, with *n* = ∣*V*∣. Using the Laplacian matrix *L*, Δ_*D*_ applied to $\vec{f}$ can be written as

$$\begin{array}{c} \Delta_{D}\vec{f}=-L\vec{f}\;. \end{array}$$ (14)

In the following we present four different approaches to discretize the Laplace-Beltrami operator, a graph-theoretical approach and three geometric approaches.

First, we look at the graph-theoretical approach, where the coordinates of the positions of the vertices are not considered. The topological Laplacian results from Eq (10) by using $w(i,x)=b_{i}^{-1}=1$, see also [24, 26, 40]. The graph-theoretical approach will be referred to as TL later in the text.

Second, for inhomogeneous triangular meshes, where the distances between vertices and the sizes of angles and triangles are different, the weighting function *w* has to be adapted according to the mesh geometry. In these approaches, the positions of the vertices are also considered. They are referred to as geometric approaches. There are different approaches to treat inhomogeneous meshes.

The first possibility is to use the Euclidean distance of adjacent vertices raised to the power of a value *α*. For Eq (10) the coefficients $b_{i}^{-1}=1$ and *w*(*i*, *x*) = ‖*e*_*ix*_‖^*α*^ are chosen. A common choice is to use the inverse of the Euclidean distance with *α* = −1 [40, 43]. This approach will be referred to later as IE.

The second approach for a geometric discretization of the Laplace-Beltrami operator is derived by minimizing the Dirichlet energy for a triangulated mesh [44, 45]. It uses cotangent weights with

$$\begin{array}{c} w\left(i,x\right)=\frac{1}{2}(\cot\left(\alpha_{ix}\right)+\cot\left(\beta_{ix}\right))\;, \end{array}$$ (15)

with the two angles *α*_*ix*_ and *β*_*ix*_ opposed to the edge *e*_*ix*_, see Fig 1(b). For edges on the boundary of the mesh, the term cot(*β*_*ix*_) is omitted, which leads to Neumann BC. A drawback of using the cotangent weights is that the value representing the integral of the Laplacian over a 1-ring neighborhood (area of the *i* ^⋆^-neighborhood) is assigned to a point sample [26]. To resolve this issue and to guarantee the correspondence between the continuous and the discrete approaches, the weights in Eq (15) are divided by the area $A_{i}^{\text{B}}$ of the barycell for the vertex *v*_*i*_ [46], resulting in

$$\begin{array}{c} w\left(i,x\right)=\frac{1}{2A_{i}^{B}}(\cot\left(\alpha_{ix}\right)+\cot\left(\beta_{ix}\right))\;. \end{array}$$ (16)

The barycell for a vertex *v*_*i*_ is framed by a polygonal line that connects the geometric centroids of triangles in *i* ^▿^ and the midpoints of the adjoined edges *e*_*ix*_, see Fig 1(c). The area of the *i* ^⋆^-neighborhood for a vertex *v*_*i*_, which is the area of the triangles that are enclosed by the vertices *v*_*x*_ ∈ *i* ^⋆^, is referred to as $A_{i}^{1}$. Then $A_{i}^{\text{B}}$ can be determined by $A_{i}^{\text{B}}=\frac{1}{3}A_{i}^{1}$. For the discretizations using the cotangent weighted formulation, the parameter $b_{i}^{-1}$ in Eq (10) is set to $b_{i}^{-1}=1$. This approach, using cotangent weights will be referred to as COT later in the manuscript.

The third geometric approach to discretize the Laplace-Beltrami operator is the finite element method (FEM), which is related to the approach using cotangent weights. Assuming that the function *f* is piecewise linear and defined by its values *f*_*i*_ on the vertices *v*_*i*_ of a triangular mesh, *f* can be interpolated using nodal basis functions *ψ*_*i*_

$$\begin{array}{c} f=\sum_{i=1}^{\left|V\right|}f_{i}\;\psi_{i}\;. \end{array}$$ (17)

We use the hat function for *ψ*_*i*_, with

$$\begin{array}{c} \psi_{i}\left(j\right)=\{\begin{array}{cc} 1 & \text{if}\;i=j\\ 0 & \text{otherwise}\;. \end{array} \end{array}$$ (18)

For two functions *f* and *g* defined on *M*, a scalar product is given by

$$\begin{array}{c} \int_{M}fg\;\text{d}a=\sum_{i=0}^{\left|V\right|}\sum_{j=0}^{\left|V\right|}f_{i}g_{i}\int_{M}\psi_{i}\psi_{j}\;\text{d}a={\left\langle \vec{f},\vec{g}\right\rangle }_{B}\;, \end{array}$$ (19)

with the area element d*a* on *M* and the mass matrix *B*. The sparse mass matrix *B* is given by

$$\begin{array}{c} B_{ij}=\int_{M}\psi_{i}\psi_{j}\;\text{d}a\;. \end{array}$$ (20)

For the FEM approach using hat functions, the elements of *B* can be calculated by

$$\begin{array}{c} B_{ij}=\{\begin{array}{cc} (\sum_{t\in i^{▿}}\left|t\right|)/6 & \text{if}\;i=j\\ (|t_{a}\left|+\right|t_{b}|)/12 & \text{if}\;e_{ij}\in E\\ 0 & \text{otherwise}\;, \end{array} \end{array}$$ (21)

where *t*_*a*_ and *t*_*b*_ are the two triangles adjacent to the edge *e*_*ij*_, see Fig 1(b). For the FEM discretization of the Laplace-Beltrami operator also a stiffness matrix *S* has to be calculated. The elements of *S*_*ij*_ can be estimated using the Eqs (12) and (15), compare [26, 47, 48].

To simplify the FEM approach, the mass matrix *B* can be replaced by a lumped mass matrix $\hat{B}$, which is defined as

$$\begin{array}{c} {\hat{B}}_{ij}=\{\begin{array}{cc} (\sum_{t\in i^{▿}}\left|t\right|)/3 & \text{if}\;i=j\\ 0 & \text{otherwise}\;. \end{array} \end{array}$$ (22)

The simplified FEM approach, which uses a lumped mass matrix $\hat{B}$, is equivalent to the method using cotangent weights presented in Eq (16), see [48].

#### Properties of Discrete Laplace-Beltrami Operators and their Eigensystems

Desirable properties of the discrete Laplacian *L* are symmetry, positive weights, positive semi-definiteness, locality, linear precision and convergence [38]. The symmetry *L*_*ij*_ = *L*_*ji*_ leads to real eigenvalues and orthogonal eigenvectors. Positive weights *w*(*i*, *j*) ≥ 0 assure, together with the symmetry, the positive semi-definiteness of *L*. The locality of the discrete Laplace-Beltrami operator enables the determination of weights *w*(*i*, *j*) using the *i* ^⋆^-neighborhood of a vertex *v*_*i*_, with *w*(*i*, *j*) = 0, if *e*_*ij*_ ∉ *E*. The linear precision implies for a linear function *f* defined on vertices *v*_*i*_ that $\Delta_{D}{\vec{f}}_{i}=0$ applies, which ensures the exact recovery of *f* from the samples. The convergence property provides the convergence from the discrete to the continuous Laplace-Beltrami operator Δ_*D*_ → Δ for a sufficient refinement of the mesh.

The Laplacian matrix *L* for the TL and the IE approach are positive semi-definite, symmetric and use positive weights. The COT and the FEM approach do not fulfill the positive weight property, if the mesh contains triangles with interior angles in the interval (*π*/2, *π*), for which the cotangent is negative. The TL approach is no geometric discretization, because it violates the linear precision and the convergence property. In contrast, the COT and the FEM approach are geometric discretizations as they fulfill the linear precision and the convergence property, but they violate the symmetry property. None of the presented discretization methods fulfill all desirable properties, see also [38].

The discrete Laplacian eigenvalue problem for a real and symmetric Laplacian matrix is given by

$$\begin{array}{c} L\;{\vec{x}}_{i}=\lambda_{i}\;{\vec{x}}_{i}\;, \end{array}$$ (23)

with eigenvectors ${\vec{x}}_{i}$ and eigenvalues *λ*_*i*_ of *L*. The Laplacian matrix *L* is real and symmetric for the TL, the IE and the COT approach. Because *L* is real and symmetric, eigenvalues *λ*_*i*_ ∈ ℝ with *λ*_*i*_ ≥ 0 are obtained. The eigenvectors ${\vec{x}}_{i}$ are real-valued and form a harmonic orthonormal basis. The corresponding eigenvalues *λ*_*i*_ can be considered as spatial frequencies. The eigenvectors ${\vec{x}}_{i}$ can be used for a spectral analysis of functions defined on the mesh *M*. The projection of a discrete function $\vec{f}$ defined on *M* onto the basis of spatial harmonic functions is performed by the inner product for Euclidean *n*-spaces $\left\langle \vec{f},{\vec{x}}_{i}\right\rangle$. For the matrix *X*, where the eigenvectors ${\vec{x}}_{i}$ represent the columns,

$$\begin{array}{c} X=[{\vec{x}}_{1}\;{\vec{x}}_{2}\cdots {\vec{x}}_{n}]\;, \end{array}$$ (24)

it applies

$$\begin{array}{c} X^{⊤}X=I\;, \end{array}$$ (25)

with the identity matrix *I*.

For the FEM formulation, the BF ${\vec{y}}_{i}$ are computed by solving the generalized symmetric definite eigenproblem

$$\begin{array}{c} S{\vec{y}}_{i}=\lambda_{i}B{\vec{y}}_{i}\;. \end{array}$$ (26)

Thus, the inversion of the mass matrix *B* is avoided. Because *B* ^−1^ *S* is not symmetric, the eigenvectors ${\vec{y}}_{i}$ are real-valued, but not orthonormal with respect to the inner product for Euclidean *n*-spaces ⟨.⟩. To use these eigenvectors as BF, the inner product, defined in Eq (19), has to be used

$$\begin{array}{c} {\left\langle \vec{f},{\vec{y}}_{i}\right\rangle }_{B}={\vec{f}}^{\;⊤}B\;{\vec{y}}_{i}\;, \end{array}$$ (27)

which assures the *B*-orthogonality, compare also [48]. The eigenvectors computed by the FEM approach can be normalized by using the *B*-relative norm

$$\begin{array}{c} {\vec{\tilde{y}}}_{i}=\frac{{\vec{y}}_{i}}{{∥{\vec{y}}_{i}∥}_{B}}\;\text{with}\;{∥{\vec{y}}_{i}∥}_{B}=\sqrt{{\left\langle {\vec{y}}_{i},{\vec{y}}_{i}\right\rangle }_{B}}\;. \end{array}$$ (28)

For a matrix $\tilde{Y}$, where the normalized eigenvectors ${\vec{\tilde{y}}}_{i}$ represent the columns

$$\begin{array}{c} \tilde{Y}=\left[{\vec{\tilde{y}}}_{1}\;{\vec{\tilde{y}}}_{2}\cdots {\vec{\tilde{y}}}_{n}\right]\;, \end{array}$$ (29)

it applies

$$\begin{array}{c} {\tilde{Y}}^{⊤}B\tilde{Y}=I\;. \end{array}$$ (30)

### Discrete spatial harmonic signal processing framework

#### Requirements

To use the eigenvectors of the discrete Laplacian Beltrami operator in the context of a signal processing framework, it is necessary that they exhibit certain properties. The eigenvectors have to form a set of BF. An inner product for the decomposition and for the reconstruction of the data has to be defined, which is used for the transformation into the domain of the spatial frequencies and for the back-transformation into the spatial domain. To be utilized for practical applications, the transformation from the spatial domain into the spatial frequency domain has to have linear properties and should fulfill Parseval’s theorem.

#### Basis functions

A complete set of linearly independent vectors can be used as basis. For real and symmetric Laplacian matrices *L* the orthonormality of the eigenvectors is given inherently, see Eqs (23) and (25). For the FEM approach, the orthonormality of the eigenvectors is assured explicitly, see Eqs (26)–(30). The property of orthogonality includes the linear independence. To use the eigenvectors as BF, they must further fulfill the property of completeness. The completeness can be shown by the dimension theorem for vector spaces. The dimensionality is equal for both the spatial representation and the representation in the spatial frequency domain. For a mesh with *n* vertices, *n* unit impulse functions are used as BF for the spatial representation. For the same mesh, we obtain *n* discrete spatial harmonic functions (eigenvectors) for the representation using spatial frequencies. The calculated eigenvectors are orthonormal and complete; therefore, they can be used as orthonormal BF.

#### Decomposition and reconstruction

For the decomposition of discrete data defined on the vertices of the triangular mesh, the inner product is used (transformation from spatial domain to spatial frequency domain). For a decomposition using the eigenvectors of a symmetric Laplacian matrix *L* (TL, IE and COT), the vector space inner product is applied. The coefficient *c*_*i*_ for a single spatial harmonic BF ${\vec{x}}_{i}$ can be determined by

$$\begin{array}{c} c_{i}=\left\langle \vec{f},{\vec{x}}_{i}\right\rangle \;. \end{array}$$ (31)

The transformation from the spatial into the spatial frequency domain is computed by

$$\begin{array}{c} {\vec{c}}^{\;⊤}={\vec{f}}^{\;⊤}\;X\;. \end{array}$$ (32)

For a decomposition using eigenvectors computed by the FEM approach, the inner product that assures the *B*-orthogonality needs to be applied

$$\begin{array}{c} c_{i}={\left\langle \vec{f},{\vec{\tilde{y}}}_{i}\right\rangle }_{B}={\vec{f}}^{\;⊤}B\;{\vec{\tilde{y}}}_{i}\;. \end{array}$$ (33)

The transformation from the spatial into the spatial frequency domain is then be computed by

$$\begin{array}{c} {\vec{c}}^{\;⊤}={\vec{f}}^{\;⊤}B\;\tilde{Y}\;. \end{array}$$ (34)

Discrete data are reconstructed using the linear combination of the coefficients *c*_*i*_ and the corresponding BF ${\vec{x}}_{i}$ or ${\vec{\tilde{y}}}_{i}$

$$\begin{array}{c} \vec{f}=\sum_{i=1}^{n}c_{i}\;{\vec{x}}_{i} \end{array}$$ (35)

or

$$\begin{array}{c} {\vec{f}}^{\;⊤}={\vec{c}}^{\;⊤}\;{\tilde{Y}}^{\;⊤}\;. \end{array}$$ (36)

#### Linearity

The property of linearity is required for many applications in which a spatial harmonic decomposition is performed. Eq (32) describes a linear transformation from the spatial into the spatial frequency domain by using the transformation matrix *X*. For two transformations

$$\begin{array}{cc} {\vec{f}}_{1}^{\;⊤} & ⟷{\vec{c}}_{1}^{\;⊤}={\vec{f}}_{1}^{\;⊤}X\\ {\vec{f}}_{2}^{\;⊤} & ⟷{\vec{c}}_{2}^{\;⊤}={\vec{f}}_{2}^{\;⊤}X\;, \end{array}$$ (37)

with the functions in the spatial domain, ${\vec{f}}_{i}$, and functions in spatial frequency domain, ${\vec{c}}_{i}$, and scalar values *a*_1_ and *a*_2_, it follows

$$\begin{array}{cc} (a_{1}{\vec{f}}_{1}^{\;⊤}+a_{2}{\vec{f}}_{2}^{\;⊤})X & =a_{1}{\vec{f}}_{1}^{\;⊤}X+a_{2}{\vec{f}}_{2}^{\;⊤}X\\ & =a_{1}{\vec{c}}_{1}^{\;⊤}+a_{2}{\vec{c}}_{2}^{\;⊤}\;. \end{array}$$ (38)

The property of linearity can be shown analogously for transformations corresponding to Eq (34) using the operator $B\tilde{Y}$.

#### Parseval’s theorem

The energy representations of a signal in the spatial domain and in the spatial frequency domain are equivalent; they must possess the same energy (Parseval’s theorem). For a discrete real-valued function $\vec{f}$ defined in the spatial domain and for its representation $\vec{c}$ in the spatial frequency domain, which was calculated by the eigenvectors of a real and symmetric Laplacian matrix *L* (TL, IE and COT), it applies

$$\begin{array}{c} \langle \vec{f},\vec{f}\rangle =\langle \vec{c},\vec{c}\rangle \;. \end{array}$$ (39)

When using the FEM approach, where the *B*-relative inner product has to be employed to compute the signal energy in the spatial domain, it applies

$$\begin{array}{c} {\left\langle \vec{f},\vec{f}\right\rangle }_{B}=\langle \vec{c},\vec{c}\rangle \;. \end{array}$$ (40)

### Numerical considerations

The Laplacian operator *L* is a sparse square matrix *n* × *n*, where *n* is the number of vertices. In the case of a symmetric Laplacian matrix (TL, IE and COT), the eigenvalues and eigenvectors of *L* are computed using a parallel implementation of the Lapack routine Dsyevr [49]. Dsyevr consists of the two subroutines Dsytrd and Dstemr. The Dsytrd-routine performs a Householder tridiagonalization of *L* and has a complexity of *O*(*n* ^3^) [50]. The second subroutine Dstemr computes the eigenvalues and eigenvectors of the resulting real symmetric tridiagonal matrix and has a complexity of *O*(*n* ^2^) [51]. Thus, the computational complexity of Dsyevr is *O*(*n* ^3^).

For asymmetric Laplacian matrices *L* (FEM approach) the eigenvalues and eigenvectors are computed using the Lapack routine Dgeev, which has a complexity of *O*(*n* ^3^). To compute the eigenvalues and eigenvectors, this method performs the reduction to an upper Hessenberg form (Dgehrd) and a Schur factorization (Dhseqr) [49].

For the FEM approach, a symmetric definite eigenproblem has to be solved. Thus, the Lapack routine Dsygv can be applied. By using a Cholesky factorization of the Laplacian matrix, the problem is reduced to a standard symmetric eigenproblem [49]. The complexity of Dsygv is also *O*(*n* ^3^). When considering the partial eigenproblem only and by using multigrid approaches and preconditioning, the computational complexity of the FEM approach can be reduced to *O*(*n* log *n*) [52]. It should be noted that two matrices have to be computed in the FEM approach for the data decomposition, since the mass matrix *B* is also required for the inner product ⟨.⟩_*B*_.

The inner product is used for the decomposition of the data defined on *n* spatial sample points. For a single BF, the complexity of computing the inner product is *O*(*n*); *n* multiplications and *n* − 1 additions of floating point numbers are carried out. The multiplications are independent; the additions can be cascaded. The complete decomposition of the data using all *n* normalized BF is performed by the multiplication of a vector containing the data and a matrix, where the columns are the normalized BF. The complexity is *O*(*n* ^2^).

### Spatial decomposition methods used for comparison with Sphara

As mentioned in the introduction, several methods for spatial decomposition of multichannel data are already available. In order to assess the performance, we have compared Sphara with PCA and ICA. When using Sphara, EEG data are decomposed by projection into the space of basis functions. This is similar to PCA or ICA, where projections into the space of principal or independent components are used. For the comparison, we utilized an own implementation of PCA and the Infomax-based ICA [8].

## Data

We illustrate the application of our proposed method to data sets that originate from a previously performed EEG experiment addressing the cortical activation related to somatosensory-evoked potentials (SEP). Eleven healthy male volunteers, aged between 26 and 40 years (mean 30.7 ± 4.3 years), ten right-handed and one left-handed, participated in the experiment. No volunteer suffered from neurological disease, and all participants were free of medication. The study was approved by the Ethics Committee of the medical faculty of the Friedrich Schiller University Jena and written informed consent was obtained. The median nerve of the right forearm was stimulated by bipolar electrodes (stimulation rate: 3.7 Hz, interstimulus interval: 270 ms, stimulation strength: motor plus sensor threshold [53, 54], constant current rectangular pulse wave impulses with a length of 50 *μ*s, number of stimulations: 6000).

**Fig 2:**
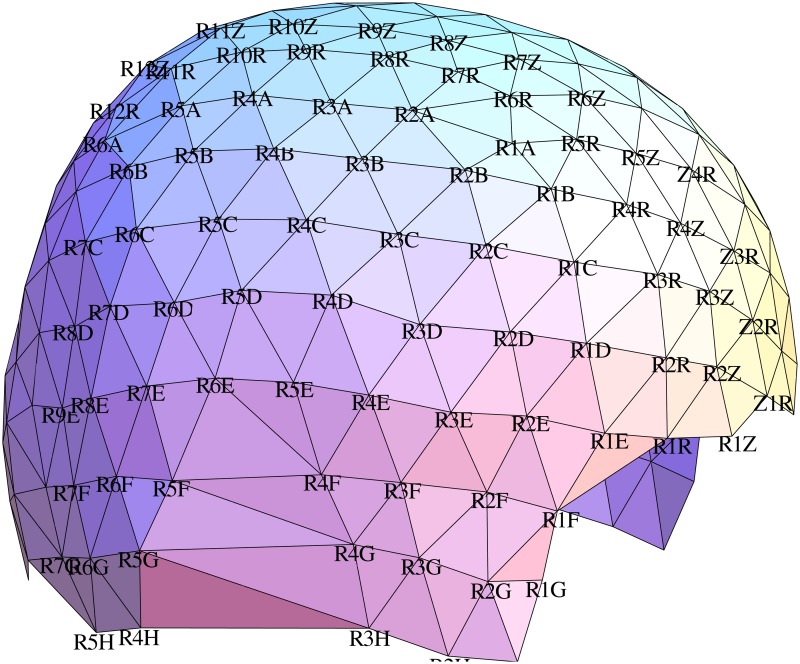
The digitized electrode positions of the EEG cap for one volunteer. The view direction is from the right side.

**Fig 3:**
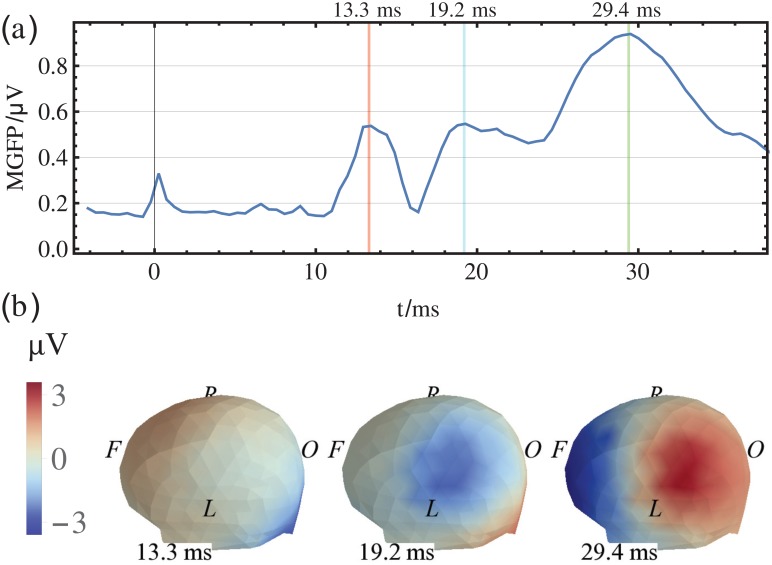
Averaged SEP data for one volunteer. (a) The mean global field power (MGFP), local maxima in activity at 13.3, 19.2 and 29.4 ms after stimulus; (b) The potential distribution on the scalp, view direction is from the top left (L: left; R: right; F: frontal; O: occipital).

**Fig 4:**
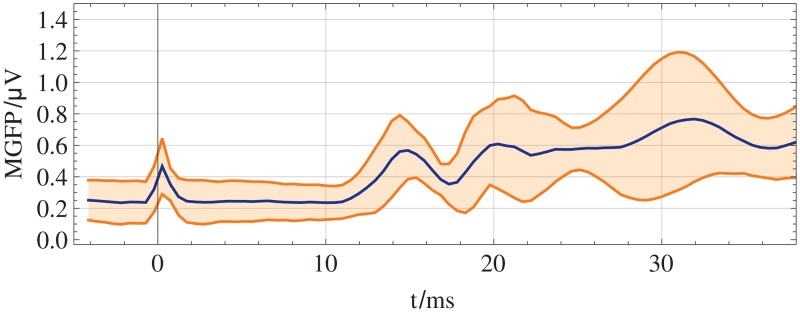
Grand average of SEP data. The grand average of the MGFP is marked by the blue line. The range of the grand average ± the standard deviation at the time sample is orange shaded.

The positions of the EEG electrodes were digitized using an optical ANT Neuro BV (ant-neuro.com) xensor 3D electrode digitizer system, see Fig 2. EEG signals were recorded using an ANT Neuro BV waveguard 256-channel EEG cap with an equidistant electrode layout and two cascaded RefaExt 128 channel amplifiers and transformed to common average reference. Data were sampled at 2048 Hz and software high-pass (24 dB/oct, cutoff-frequency 2 Hz) and notch (50 Hz and two harmonics) filtered. All trials were manually checked for artifacts, the remaining trials were averaged, see S1 Dataset. The mean global field power (MGFP) and topographic maps of the averaged and filtered SEP data of a single volunteer at selected points in time are shown in Fig 3. In summary, the grand average (GA) and the range of ± the standard deviation of the MGFP of all subjects is shown in Fig 4. The exact points in time of the maximum potentials vary between volunteers. For each volunteer, the specific time of the P14, N20 and N30 potentials are manually estimated.

To demonstrate the ability of the noise suppression of Sphara, Gaussian white noise was added to averaged SEP data. The following signal-noise-ratios (SNR) were used (dB): 10, 6, 3, 0, -3, -6, -10. In the simulation, the average SEP data were used as reference (signal). For each SNR 100 000 noise realizations were analyzed.

## Results

**Fig 5:**
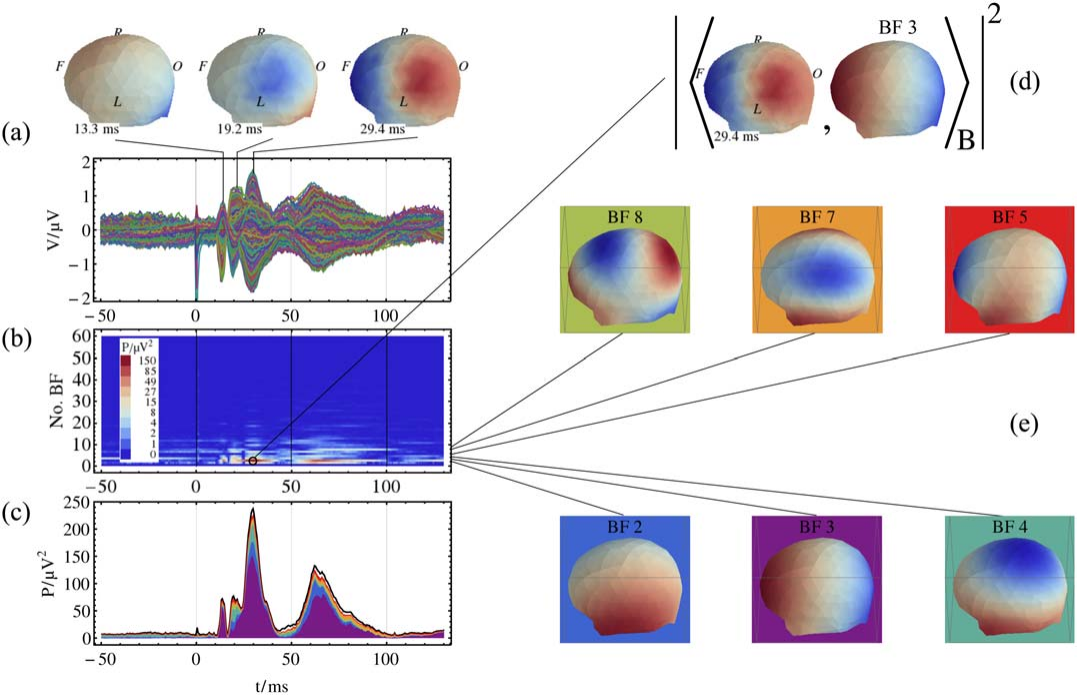
The spatial harmonic decomposition of the SEP data for one volunteer. (a) Averaged trials of the SEP data, 256 channels and the potential distribution for P14, N20, and N30 are shown. The view direction is top left. (b) Time-spatial-harmonic representation of the power of the SEP data using 60 BF with the lowest frequencies, starting with the low frequency at the bottom. (c) Contribution of selected BF to the MGFP of the SEP data. The MGFP is represented by the black line. The contribution of the BF is color-coded in the background, compare subfigure (e). (d) For each point of the time-spatial-harmonic representation, the square of the absolute value of the *B*-related inner product between the potential distribution and the BF is computed. (e) Selection of BF used for the data decomposition; note the different color scaling for each BF; the color code of the background is used in subfigure (c).

The SEP data of the eleven volunteers were decomposed by using the base functions for each of the three discretization approaches. The procedure of the spatial harmonic decomposition of the SEP data for one volunteer is illustrated in Fig 5. The SEP data consist of 256 EEG-channels, which are shown in Fig 5(a). The decomposition was performed by calculating the inner product of the multichannel data and the BF, see Fig 5(d). The power contribution of the spatial harmonic BF to the SEP data at each time step is shown in Fig 5(b), which shows the time versus spatial harmonic frequency representation of the decomposed data.

**Fig 6:**
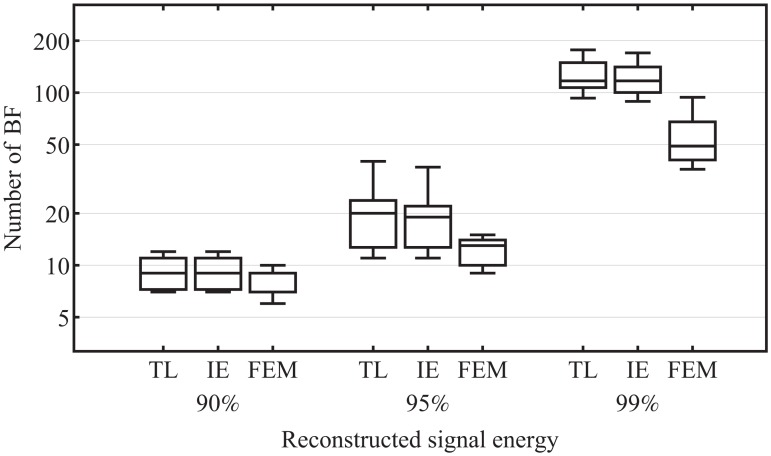
Number of coefficients that are required to achieve 90%, 95% and 99% of the total signal energy. (all samples in the time range from 50 ms before to 130 ms after stimulation). The left bars for each signal power threshold represent the results for TL, the middle bars for IE and the right bars for FEM, note the logarithmic scaling of the Y-axis.

We investigated how many of the spatial-harmonic coefficients are required to achieve 90%, 95% and 99% of the signal energy of the samples in the time range from 50 ms before to 130 ms after stimulation. We compared BF, computed by the TL, IE and FEM approaches. In the first case, the individually tracked sensor positions of the volunteers were used to determine the BF using the IE and FEM approach. The BF of the TL approach can be determined without information of the sensor positions. The BF with the largest energy contribution are used for signal reconstruction. The results are shown in Fig 6. The available compression ratios in the best, in the median and in the worst case are listed in Table 1. The compression ratio is the quotient of the maximum number of available BF and the number of BF that are used to restore the signal at a given quality. A maximum of 256 basis functions are available to reconstruct the entire signal energy. The significance of the influence of the three discretization methods on the achieved compression ratio was analyzed using the Mann-Whitney U test. This test was chosen because not all examined data are normally distributed, tested by the Pearson *χ* ^2^ test. None of the methods is significantly superior in reconstructing 90% of the signal energy. There is no significant difference between the TL and IE approach in the ability to present EEG data compactly. FEM provides significantly better results than TL and IE in the reconstruction of 95% and 99% of the signal energy, see Fig 6 and Table 2. The main contribution to the signal power of the SEP data is provided by the low frequency spatial-harmonic BF as exemplarily shown in Fig 5(b).

**Table 1:** Compression ratios which can be achieved by applying Sphara to the SEP data, if 90%, 95% and 99% of the total signal energy of the SEP data of the volunteers are restored.

	90%			95%			99%		
	TL	IE	FEM	TL	IE	FEM	TL	IE	FEM
Best case	36.6	36.6	42.7	23.3	23.3	28.4	2.8	2.9	7.1
Median case	28.4	28.4	36.6	12.8	13.5	23.3	2.2	2.2	2.9
Worst case	21.3	21.3	25.6	6.4	6.9	17.1	1.4	1.5	2.7

**Table 2:** Statistical evaluation of the compression of the SEP data, *p*-values of the statistical evaluation of the compression of the SEP data, using the Mann-Whitney U test.

	TL vs. IE	TL vs. FEM	IE vs. FEM
90%	0.973236	0.096671	0.096671
95%	0.620494	0.009212	0.009192
99%	0.490152	0.000081	0.000106

**Fig 7:**
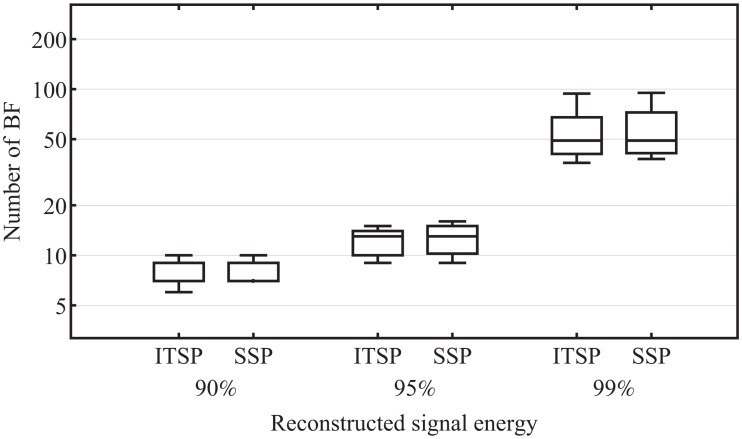
Individually tracked sensor positions (ITSP) vs. standard sensor positions (SSP), omparison of the effect on the compact representation of the EEG data. In the median case, for both ITSP and SSP the same number of BF are necessary to achieve 90%, 95% and 99% of the total signal energy.

For the FEM approach, we have calculated two sets of BF, first using individually tracked sensor positions (ITSP) and second using standard sensor positions (SSP) provided by the manufacturer of the EEG cap. There is no difference between the two methods in the compact representation of the analyzed EEG data. In the median case, the same number of BF are necessary to achieve 90%, 95% and 99% of the total signal energy, see Fig 7.

**Fig 8:**
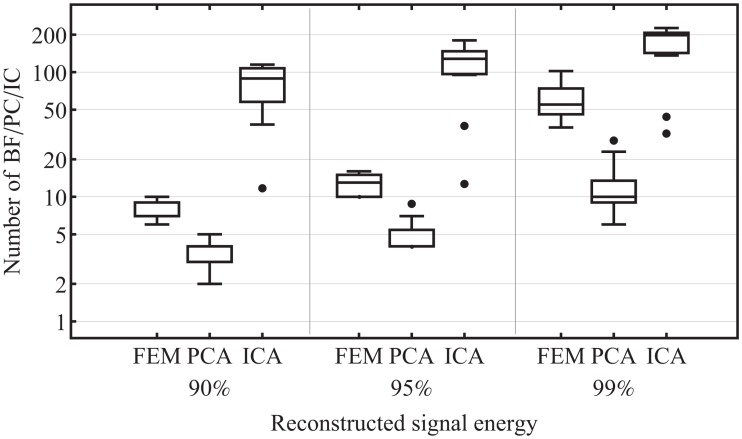
FEM vs. PCA vs. ICA. The comparison of the effect on the compact representation of the investigated EEG data.

We compared the FEM approach with PCA and ICA regarding the ability of a compact representation of EEG data in a spatial aspect. In PCA, the PC are sorted in decreasing order by there variances, which represents their energy contribution to the investigated data. In the Infomax-based ICA, the independent components (IC) are sorted in descending order of mean projected variance. The maximum number of IC that are to be calculated is determined by the rank of the covariance matrix of the analyzed data. The number of BF, PC and IC to achieve 90%, 95% and 99% of the signal energy of the investigated EEG data are evaluated. The results are shown in Fig 8. PCA comprises clearly the best performance on the compact representation of the EEG data. The second best compression ratio achieved the Sphara approach. ICA revealed the worst performance for the investigated EEG data.

**Fig 9:**
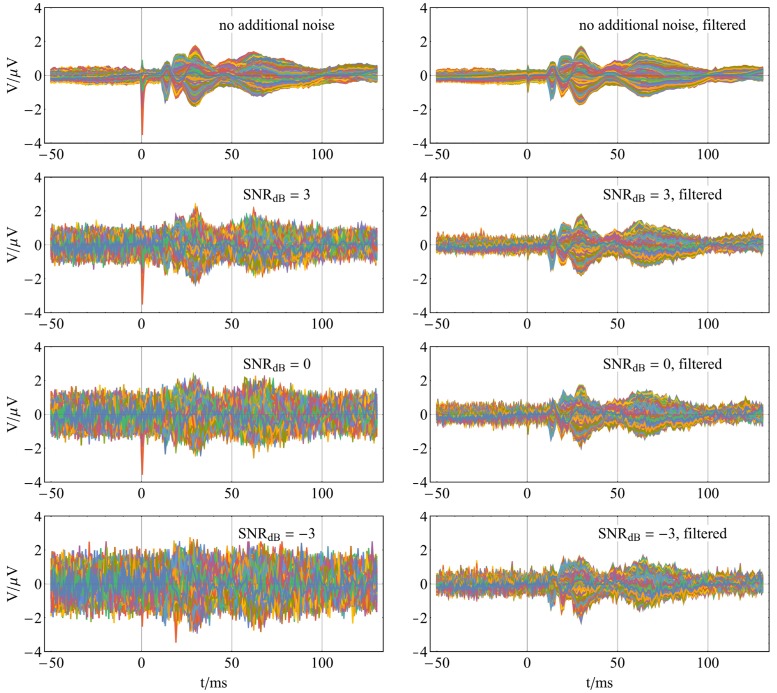
Example of noise suppression using spatial harmonic low pass filter. Left column unfiltered data, right column spatial harmonic low-pass filtered data; in the first row averaged SEP data without additional noise; in the following rows SEP data with additional Gaussian white noise with 3, 0, -3 dB signal to noise ratio.

**Fig 10:**
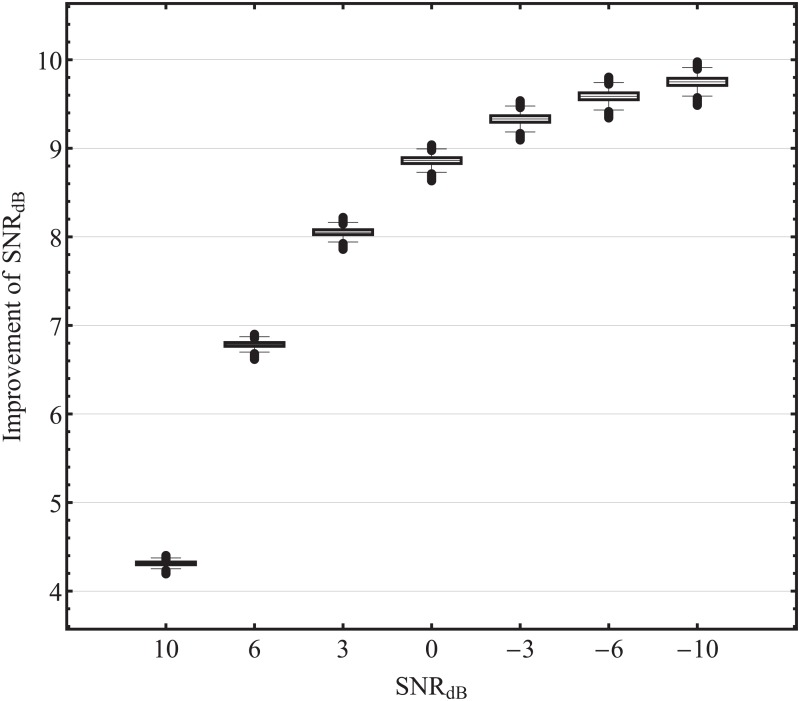
Noise suppression using spatial harmonic low pass filter. Improvement of the SNR (shown on the Y-axis) using a spatial harmonic low-pass filter applied to data with different noise ratios (shown on the X-axis).

The capability of the new method for noise suppression is demonstrated by implementing a spatial low pass filter. For this purpose, we designed a filter with 25 low-frequency basis functions. Depending on the data of the volunteers, between 96.4 and 98.6% of the signal energy of the EEG data can be reconstructed with 25 low-frequency basis functions. In the simulation, performed to investigate the noise reduction, Gaussian white noise with different noise ratios was added to the averaged SEP data. Subsequently, the noisy data were low-pass filtered with Sphara. An example of the averaged SEP data, with additional Gaussian white noise and the results of the spatially harmonic low-pass filtering are shown in Fig 9. In a simulation with 100 000 repetitions, the ability of the spatial harmonic analysis for noise reduction could be shown. The improvement of the SNR by spatially harmonic low-pass filtering is in the median case between 4.31 dB and 9.74 dB, see Fig 10.

## Discussion

The eigensystem of the discrete Laplace-Beltrami operator defined on a triangular mesh forms a set of harmonic orthonormal BF, which can be adaptively computed using the topology of the sensor setup and the sensor positions in ℝ^3^. The set of BF enables the spatial Fourier analysis of arbitrarily arranged multi-sensor data.

Activations in the human brain cause electrical potentials around the entire head. For anatomical reasons, it is difficult or impossible to observe the electrical potential around the whole head. In EEG measurements, the head surface is only partially covered by sensors, therefore only a part of the electrical potentials that emerge at the surface of the human head are captured. The potential on the boundary of the examined area of the surface are not zero or constant. For this reason, Neumann boundary conditions are more appropriate to compute the BF.

For the data analysis described in this article, three discretization schemes are used to compute the BF. The three discretization methods require different information about the triangular mesh for the calculation of the BF. Also the effort of calculating the BF and of the decomposition of the EEG data is different. For the determination of BF by means of TL, only the layout of the triangle mesh must be known. The particular information about coordinates of the sensor positions are not necessary. For different EEG cap sizes with the same triangulation, the same BF can be used. These BF can be already determined during the design process of the EEG cap. For the calculation of the BF using the IE and the FEM approach, additional information about the sensor positions are necessary. The eigensystem of the discrete Laplace-Beltrami operator needs to be computed only once for a EEG electrode setup, if the electrode positions are not individually determined. If the electrode positions are individually tracked for each volunteer, the BF have to be determined for each measurement session. In contrast to TL and IE, the Laplace-Beltrami operator using the FEM approach represents a geometric discretization and fulfills the convergence property. It converges to the continuous solution for a sufficient refinement of the mesh. Subsequently, the decomposition of the recorded multi-sensor data for a single point in time is carried out by a multiplication of the matrix with the BF and a vector with the measurement data. For the data decomposition using the FEM approach, in addition to the matrix of the BF the mass matrix is required and the computational effort is slightly increased compared to TL and IE.

We compared TL, IE and FEM with regard to their applicability for a compact representation of EEG data. When reconstructing 90% of the signal energy of the EEG data, none of the discretization methods is superior. Using the FEM approach, a significantly more compact representation of data can be achieved, in the case of 95% and 99% reconstructed signal energy. However, compared to TL and IE it requires a slightly higher effort for the determination of the BF and the data decomposition.

We determined the BF for IE and FEM using for each volunteer individually tracked and standard sensor positions, and we compared the influence on the compact representation of the EEG data. For the considered data, no differences were observed in the median case. However, there are applications conceivable in which individually tracked sensor positions are advantageous, e. g. approaches that incorporate individual volume conductor models of the heads of volunteers. This can be the field of future investigations.

In terms of compact representation of the EEG data, PCA has performed better and ICA worse than Sphara. The better performance of PCA can be explained by the fact, that the maximization of the energy, represented by the principal components, is a design criterion of PCA. To achieve this, the measured time series need to be analyzed. ICA is a decomposition method, which splits the multivariate data into statistically independent components. These statistically independent components do not necessarily have a high energy content. For most multivariate decomposition methods, such as PCA and ICA, the recorded multivariate time series are employed to generate the components for a spatial decomposition. In contrast to these methods, in Sphara the BF for the spatial decomposition are generated using topological information of the electrode arrangement and the sensor positions. Therefore, the BF can be determined prior to the recording of the time series. This is particularly beneficial for online data processing and for other time critical applications.

The ability of the new approach to denoise EEG data is demonstrated by averaged SEP data with additional artificial noise. Utilizing the Sphara BF a spatial low-pass filter was implemented. Depending on the amount of added artificial noise, an median improvement of SNR between 4.31 dB and 9.74 dB could be achieved by the spatial low-pass filtering. The filtering is done only in the spatial domain. Different time samples of the EEG data are considered separately. This facilitates the online denoising of EEG data.

The Sphara approach can also be combined with frequency and time-frequency analysis methods. Furthermore, the spatial-harmonic approach can be applied to various fields where multi-sensor data are measured on surfaces and manifolds in *R* ^3^.

In most multivariate decomposition methods, such as PCA, ICA and PARAFAC, the recorded multivariate time series are employed to generated components for a spatial decomposition. In contrast to these methods, in Sphara the BF for the spatial decomposition are generated using topological information of the electrode arrangement and the sensor positions solely. Therefore, the BF can be determined prior to the recording of the time series.

## Supporting Information

S1 Dataset SEP data that has been analyzed. The data set contains averaged and filtered SEP data of the volunteers as well as the standard electrode positions and triangle list of the ANT Neuro BV waveguard 256-channel EEG cap.
(ZIP)

## References

[1] Rao K, Kim D, Hwang J (2010) Fast Fourier Transform: Algorithms and Applications Signals and communication technology. Springer.

[2] Bashashati A, Fatourechi M, Ward RK, Birch GE (2007) A survey of signal processing algorithms in brain-computer interfaces based on electrical brain signals. Journal of Neural Engineering 4: R32–R57. 10.1088/1741-2560/4/2/R03 17409474

[3] Brunner C, Naeem M, Leeb R, Graimann B, Pfurtscheller G (2007) Spatial filtering and selection of optimized components in four class motor imagery EEG data using independent components analysis. Pattern Recognition Letters 28: 957–964. 10.1016/j.patrec.2007.01.002

[4] Pourtois G, Delplanque S, Michel C, Vuilleumier P (2008) Beyond conventional event-related brain potential (ERP): Exploring the time-course of visual emotion processing using topographic and principal component analyses. Brain Topography 20: 265–277. 10.1007/s10548-008-0053-6 18338243

[5] Soong ACK, Koles ZJ (1995) Principal-component localization of the sources of the background EEG. IEEE Transactions on Biomedical Engineering 42: 59–67. 10.1109/10.362918 7851931

[6] Koles ZJ, Lind JC, Soong ACK (1995) Spatio-temporal decomposition of the EEG: a general approach to the isolation and localization of sources. Electroencephalography and clinical Neurophysiology 95: 219–230. 10.1016/0013-4694(95)00083-B 8529553

[7] Lagerlund TD, Sharbrough FW, Busacker NE (1997) Spatial filtering of multichannel electroencephalographic recordings through principal component analysis by singular value decomposition. Journal of Clinical Neurophysiology 14: 73–82. 10.1097/00004691-199701000-00007 9013362

[8] Bell A, Sejnowski T (1995) An information-maximization approach to blind separation and blind deconvolution. NEURAL COMPUTATION 7: 1129–1159. 10.1162/neco.1995.7.6.1129 7584893

[9] Hyvärinen A, Oja E (2000) Independent component analysis: algorithms and applications. Neural Networks 13: 411–430. 10.1016/S0893-6080(00)00026-5 10946390

[10] Jung TP, Makeig S, Westerfield M, Townsend J, Courchesne E, Sejnowski TJ (2001) Analysis and visualization of single-trial event-related potentials. Human Brain Mapping 14: 166–185. 10.1002/hbm.1050 11559961 PMC6871967

[11] Delorme A, Makeig S (2004) EEGLAB: an open source toolbox for analysis of single-trial EEG dynamics including independent component analysis. Journal of Neuroscience Methods 134: 9–21. 10.1016/j.jneumeth.2003.10.009 15102499

[12] Hyvärinen A, Karhunen J, Oja E (2001) Independent Component Analysis. Wiley-Interscience, 1st edition.

[13] De Lucia M, Michel CM, Murray M (2010) Comparing ica-based and single-trial topographic erp analyses. Brain Topography 23: 119–127. 10.1007/s10548-010-0145-y 20422446

[14] Serby H, Yom-Tov E, Inbar GF (2005) An improved P300-based brain-computer interface. IEEE Transactions on Neural Systems and Rehabilitation Engineering 13: 89–98. 10.1109/TNSRE.2004.841878 15813410

[15] Bayliss J, Ballard D (2000) A virtual reality testbed for brain-computer interface research. IEEE Transactions on Rehabilitation Engineering 8: 188–190. 10.1109/86.847811 10896182

[16] Miwakeichi F, Martinez-Montes E, Valdes-Sosa PA, Nishiyama N, Mizuhara H, Yamaguchia Y (2004) Decomposing EEG data into space-time-frequency components using parallel factor analysis. Neuroimage 22: 1035–1045. 10.1016/j.neuroimage.2004.03.039 15219576

[17] Morup M, Hansen LK, Herrmann CS, Parnas J, Arnfred SM (2006) Parallel factor analysis as an exploratory tool for wavelet transformed event-related EEG. Neuroimage 29: 938–947. 10.1016/j.neuroimage.2005.08.005 16185898

[18] Cichocki A, Washizawa Y, Rutkowski T, Bakardjian H, Phan AH, Choi S, et al (2008) Noninvasive BCIs: Multiway signal-processing array decompositions. Computer 41: 34–42. 10.1109/MC.2008.431

[19] Durka PJ, Blinowska KJ (1995) Analysis of EEG transients by means of matching pursuit. Annals of Biomedical Engineering 23: 608–611. 10.1007/BF02584459 7503462

[20] Durka P, Matysiak A, Martinez Montes E, Valdes Sosa P, Blinowska K (2005) Multichannel matching pursuit and EEG inverse solutions. Journal of Neuroscience Methods 148: 49–59. 10.1016/j.jneumeth.2005.04.001 15908012

[21] Gratkowski M, Haueisen J, Arendt-Nielsen L, Zanow F (2007) Topographic matching pursuit of spatio-temporal bioelectromagnetic data. Przeglad Elektrotechniczny 83: 138–141.

[22] Gratkowski M, Haueisen J, Arendt-Nielsen L, Chen ACN, Zanow F (2008) Decomposition of biomedical signals in spatial and time-frequency modes. Methods of Information in Medicine 47: 26–37. 18213425 10.3414/me0355

[23] Wingeier BM, Nunez PL, Silberstein RB (2001) Spherical harmonic decomposition applied to spatial-temporal analysis of human high-density electroencephalogram. Physical Review E 64: 14941–14962. 10.1103/PhysRevE.64.051916 11735977

[24] Chung FRK (1997) Spectral Graph Theory, volume 92. American Mathematical Society. CBMS Regional Conference Series in Mathematics.

[25] Taubin G (2000) Geometric signal processing on polygonal meshes In: Coquillart S, Duke D, editors, STAR Proceedings of Eurographics 2000. Eurographics Association.

[26] Zhang H, van Kaick O, Dyer R (2007) Spectral methods for mesh processing and analysis In: Schmalstieg D, Bittner J, editors, STAR Proceedings of Eurographics. volume 92, pp. 1–22.

[27] Reuter M, Wolter FE, Peinecke N (2006) Laplace-beltrami spectra as ‘Shape-DNA’ of surfaces and solids. Computer-Aided Design 38: 342–366. 10.1016/j.cad.2005.10.011

[28] Haines GV (1988) Computer-programs for spherical cap harmonic-analysis of potential and general fields. Computers & Geosciences 14: 413–447. 10.1016/0098-3004(88)90027-1

[29] De Santis A (1991) Translated origin spherical cap harmonic analysis. Geophysical Journal International 106: 253–263. 10.1111/j.1365-246X.1991.tb04615.x

[30] Bradshaw LA, Wikswo JP (2001) Spatial filter approach for evaluation of the surface laplacian of the electroencephalogram and magnetoencephalogram. Annals of Biomedical Engineering 29: 202–213. 10.1114/1.1352641 11310782

[31] Nunez PL, Westdorp AF (1994) The surface laplacian, high resolution EEG and controversies. Brain Topography 6: 221–226. 10.1007/BF01187712 8204409

[32] Nunez PL, Silberstein RB, Cadusch PJ, Wijesinghe RS, Westdorp AF, Srinivasan R, et al (1994) A theoretical and experimental study of high resolution EEG based on surface laplacians and cortical imaging. Electroencephalography and Clinical Neurophysiology 90: 40–57. 10.1016/0013-4694(94)90112-0 7509273

[33] Gramfort A, Clerc M (2007) Low dimensional representations of MEG/EEG data using laplacian eigenmaps. In: Joint Meeting of the 6th International Symposium on Noninvasive Functional Source Imaging of the Brain and Heart and the International Conference on Functional Biomedical Imaging. pp. 169–172.

[34] Ramirez-Velez M, Staba R, Barth DS, Meyer FG (2006) Nonlinear classification of EEG data for seizure detection. In: 3rd IEEE International Symposium on Biomedical Imaging. pp. 956–959.

[35] Graichen U, Eichardt R, Fiedler P, Strohmeier D, Haueisen J (2011) Adaptive spatial harmonic analysis of eeg data using laplacian eigenspace. In: Noninvasive Functional Source Imaging of the Brain and Heart 2011 8th International Conference on Bioelectromagnetism (NFSI & ICBEM) 2011, 8th International Symposium on. pp. 18–21.

[36] Chavel I (1984) Eigenvalues in Riemannian Geometry, volume 115 of *Pure and Applied Mathematics*. Academic Press.

[37] Rosenberg S (1997) The Laplacian on a Riemannian Manifold: An Introduction to Analysis on Manifolds Number 31 in London Mathematical Society Student Texts. Cambridge University Press.

[38] Wardetzky M, Mathur S, Kälberer F, Grinspun E (2007) Discrete laplace operators: No free lunch In: Belyaev A, Garland M, editors, SGP07: Eurographics Symposium on Geometry Processing. Eurographics Association, pp. 33–37.

[39] Berger M (2003) A Panoramic View of Riemannian Geometry. Springer.

[40] Taubin G (1995) Signal processing approach to fair surface design. In: Proceedings of the ACM SIGGRAPH Conference on Computer Graphics. pp. 351–358.

[41] Sorkine O (2006) Differential representations for mesh processing. Computer Graphics Forum 25: 789–807. 10.1111/j.1467-8659.2006.00999.x

[42] Zhang H, van Kaick O, Dyer R (2010) Spectral mesh processing. Computer Graphics Forum 29: 1865–1894. 10.1111/j.1467-8659.2010.01655.x

[43] Fujiwara K (1995) Eigenvalues of Laplacians on a closed riemannian manifold and its nets. Proceedings of the American Mathematical Society 123: 2585–2594. 10.1090/S0002-9939-1995-1257106-5

[44] Pinkall U, Polthier K (1993) Computing discrete minimal surfaces and their conjugates. Experimental Mathematics 2: 15–36. 10.1080/10586458.1993.10504266

[45] Polthier K (2002) Computational aspects of discrete minimal surfaces. In: Hass J, Hoffman D, Jaffe A, Rosenberg H, Schoen R, Wolf M, editors, Proceedings of the Clay Summer School on Global Theory of Minimal Surfaces.

[46] Meyer M, Desbrun M, Schröder P, Barr A (2003) Discrete differential geometry operators for triangulated 2-manifolds In: Hege HC, Polthier K, editors, Visualization and Mathematics III, Springer pp. 35–57.

[47] Dyer R, Zhang RH, Möller T, Clements A (2007) An investigation of the spectral robustness of mesh laplacians Technical report, Simon Fraser University, GrUVi Lab, Burnaby, Canada.

[48] Vallet B, Levy B (2007) Spectral geometry processing with manifold harmonics Technical Report inria-00186931, Université Nancy, Institut National Polytechnique de Lorraine.

[49] Anderson E, Bai Z, Bischof C, Blackford S, Demmel J, Dongarra J, et al (1999) LAPACK Users’ Guide Society for Industrial and Applied Mathematics, 3rd edition.

[50] Golub GH, Van Loan CF (1996) Matrix Computations. Johns Hopkins University Press, 3rd edition.

[51] Dhillon IS, Parlett BN (2004) Multiple representations to compute orthogonal eigenvectors of symmetric tridiagonal matrices. Linear Algebra and its Applications 387: 1–28. 10.1016/j.laa.2003.12.028

[52] Neymeyr K (2003) Solving mesh eigenproblems with multigrid efficiency. In: Kuznetsov Y, Neittaanmäki P, Pironneau O, editors, Numerical Methods for Scientific Computing. Variational problems and applications. CIMNE, Barcelona.

[53] Mauguiere F, Allison T, Babiloni C, Buchner H, Eisen AA, Goodin DS, et al (1999) Somatosensory evoked potentials In: Deuschl G, Eisen A, editors, Recommendations for the Practice of Clinical Neurophysiology: Guidelines of the International Federation of Clinical Neurophysiology, Elsevier Science B. V., chapter 2.4 pp. 79–90. 10590978

[54] Cruccu G, Aminoff M, Curio G, Guerit J, Kakigi R, Mauguiere F, et al (2008) Recommendations for the clinical use of somatosensory-evoked potentials. Clinical Neurophysiology 119: 1705–1719. 10.1016/j.clinph.2008.03.016 18486546

